# Comparative analysis of endurance, strength and body composition indicators in professional, under-23 and junior cyclists

**DOI:** 10.3389/fphys.2022.945552

**Published:** 2022-08-05

**Authors:** Lidia B. Alejo, Almudena Montalvo-Pérez, Pedro L. Valenzuela, Carlos Revuelta, Laureano M. Ozcoidi, Víctor de la Calle, Manuel Mateo-March, Alejandro Lucia, Alfredo Santalla, David Barranco-Gil

**Affiliations:** ^1^ Physical Activity and Health Research Group (PaHerg), Research Institute of Hospital 12 de Octubre (imas12), Madrid, Spain; ^2^ Faculty of Sport Sciences, Universidad Europea de Madrid, Madrid, Spain; ^3^ Caja Rural Professional Team, Navarra, Spain; ^4^ Victor de la Calle—MMR Academy Junior Team, Asturias, Spain; ^5^ Sport Science Department, Universidad Miguel Hernández, Elche, Spain; ^6^ Department of Sport and Computer Science, Section of Physical Education and Sports, Faculty of Sport, Universidad Pablo de Olavide, Sevilla, Spain

**Keywords:** predictors, performance, determinants, laboratory, testing, cycling

## Abstract

**Purpose:** To compare endurance, strength and body composition indicators between cyclists of three different competition age categories.

**Methods:** Fifty-one male road cyclists classified as either junior (*n* = 13, age 16.4 ± 0.5 years), under-23 [(U23), *n* = 24, 19.2 ± 1.3 years] or professional (*n* = 14, 26.1 ± 4.8 years) were studied. Endurance (assessed through a maximal incremental test and an 8-minute time-trial), strength/power (assessed through incremental loading tests for the squat, lunge and hip thrust exercises) and body composition (assessed through dual energy X-ray absorptiometry) were determined on three different testing sessions.

**Results:** U23 and, particularly professional, cyclists attained significantly (*p* < 0.05) higher values than juniors for most of the analyzed endurance indicators [time-trial performance, maximum oxygen uptake (VO_2max_), peak power output (PPO), respiratory compensation point (RCP), and ventilatory threshold (VT)]. Significant differences (*p* < 0.05) between U23 and professionals were also found for time-trial performance, PPO and VT, but not for other markers such as VO_2max_ or RCP. Professional cyclists also showed significantly (*p* < 0.05) lower relative fat mass and higher muscle mass levels than U23 and, particularly, juniors. No consistent differences between age categories were found for muscle strength/power indicators.

**Conclusion:** Endurance (particularly time-trial performance, PPO and VT) and body composition (fat and muscle mass) appear as factors that best differentiate between cyclists of different age categories, whereas no consistent differences are found for muscle strength/power. These findings might help in performance prediction and/or talent identification and may aid in guiding coaches in the design of training programs focused on improving those variables that appear more determinant.

## 1 Introduction

The factors that contribute to top-level performance in sports remain largely unknown (Foster et al., 2022). For instance, in road cycling different indicators have been reported as determinants of performance, notably endurance-related indicators such as the capacity to produce high power outputs even in the presence of fatigue (Van Erp et al., 2021, 2021; Mateo-March et al., 2022; Valenzuela et al., 2022) or anthropometric indicators such as a low body mass (van der Zwaard et al., 2019; Leo et al., 2021b). Achieving the top level in any sport and, particularly, in road cycling is a long-term process that takes several years and requires specific characteristics that can be improved along the career trajectory, from junior to under-23 (U23), to finally reaching the professional category. However, there is scarce evidence on the actual physiological/performance differences between cyclists of different age categories that could help identify specific characteristics that should be improved in the younger categories to increase the odds of reaching top-level performance.

As with most endurance sports, cycling performance has been reported to be mainly determined by physiological endurance indicators such as maximum oxygen uptake (VO_2max_), the so-called ‘lactate threshold’ [or other surrogates such as critical power or ventilatory threshold (VT)], and exercise economy (Joyner and Coyle, 2008; van der Zwaard et al., 2021). Indeed, strong evidence supports these laboratory-based indicators as predictors of cycling performance (Hawley and Noakes, 1992; Nichols et al., 1997; Bishop et al., 1998, 2000; Balmer et al., 2000; Bentley et al., 2001; Amann et al., 2006), and some studies have reported that indicators such as maximal aerobic capacity [represented by VO_2max_ or peak power output (PPO)] might be positively associated with the probability of a junior cyclist reaching the professional category (Svendsen et al., 2018). Controversy exists, however, on whether these endurance indicators can accurately differentiate cyclists of different age categories. Thus, whereas some authors have found a higher VO_2max_ or PPO in professional cyclists than in cyclists of lower categories [amateur (∼22 years) or junior (∼18 years)] (Pérez-Landaluce et al., 2002), have failed to find such differences (Marín-Pagán et al., 2021).

Notably, while some studies have compared the performance (Leo et al., 2022), training/competition characteristics (Leo et al., 2021a) or endurance capabilities (Pérez-Landaluce et al., 2002; Leo et al., 2021a; Marín-Pagán et al., 2021) between cyclists of different age categories, there is little or no evidence on whether other important factors such as muscle strength or body composition also differ. Accumulating evidence suggests that muscle strength/power plays a major role in cycling performance. For example, Kordi et al. recently reported that knee extension maximum voluntary torque was positively associated with both critical power and with the amount of work that can be completed above this intensity (known as W’ and considered as a marker of the so-called “anaerobic” capacity) (van der Zwaard et al., 2019; Kordi et al., 2021). Likewise, body composition has been reported to condition cycling performance. For instance, muscle mass indicators such as quadriceps volume have been identified as one of the main determinants of peak power production capacity and W’ in cyclists (Miura et al., 2002; Kordi et al., 2018, 2020a; van der Zwaard et al., 2019; Douglas et al., 2021). To the best of our knowledge, however, no previous study has determined whether strength/power or body composition indicators differ between cyclists of different age categories.

The aim of the present study was, therefore, to compare endurance, strength and body composition indicators between cyclists of three different categories (junior, U23 and professional).

## 2 Materials and methods

### 2.1 Participants

Fifty-one male road cyclists classified as junior (*n* = 13, age 16.4 ± 0.5 years), U23 (*n* = 24, 19.2 ± 1.3 years) or professionals [*Union Cycliste Internationale* (UCI) Pro-Team] (*n* = 14, 26.1 ± 4.8 years) volunteered to participate in the study. U23 and professional cyclists competed actively at national or international level (including in European or World Championships with the national team of their country of birth). All cyclists were assessed at the end of the ‘pre-season’ period, after at least 2 months had elapsed since the last competition of the previous season. To participate in the study, cyclists had to be free of musculoskeletal injuries or other conditions that could hinder their participation. They were all informed of the study procedures and provided written informed consent. The study was approved by the Ethical Committee of Alcorcón University Hospital (approval number 19/86), and all procedures were conducted following the standards established by the Declaration of Helsinki and its later amendments.

### 2.2 Study design

The study followed a cross-sectional observational design. Participants visited the laboratory on three different days interspersed by 48 h. All tests were performed at approximately the same time of the day and under the same conditions (temperature 20 ± 3 °C, humidity 25 ± 3%). Subjects were instructed to maintain their normal dietary pattern and to refrain from doing intense exercise and consuming ergogenic aids/caffeine 48 h before each testing session. The first laboratory visit consisted of body composition assessment and a maximal incremental cycling test. During their second visit, subjects completed strength tests, and during the third visit they performed a simulated 8-minute time-trial.

### 2.3 Measures

#### 2.3.1 Body composition

Height was determined using a wall stadiometer (Seca 437). Weight and body composition [whole body fat and muscle mass, and bone mineral content (BMC)] was measured by dual energy X-ray absorptiometry (DXA, Hologic QDR series Discovery; Bedford, MA). DXA assessments were performed at least 2 days after the last exercise session. Participants were recommended to maintain a similar eating and sleeping routine the day before each testing session and were advised to be euhydrated.

#### 2.3.2 Incremental cycling test

Cyclists performed a graded exercise test on their own bikes, which were placed on a validated indoor trainer (Hammer, CycleOps, Madison, WI) (Lillo-Bevia and Pallarés, 2018). They started with a standardized 10-minute warm-up at 75 W, and they subsequently accomplished a maximal incremental cycling test with an initial workload of 75 W, increasing by 5 W every 12 s (ramp-like protocol) until volitional exhaustion or when pedaling cadence could not be maintained above 60 rpm. Gas exchange data were collected breath-by-breath (Ultima Series Medgraphics, Cardiorespiratory Diagnostics, Saint Paul, MN). The VT was determined as the workload at which an increase in both the ventilatory equivalent for oxygen (VE∙VO^−1^) and end-tidal partial pressure of carbon dioxide (PetCO_2_) occurred with no concomitant increase in the ventilatory equivalent for carbon dioxide (VE∙VCO^−1^), whereas the respiratory compensation point (RCP, also termed “second ventilatory threshold”) corresponded to the work rate at which both VE∙VO^−1^ and VE∙VCO^−1^ increased together with a decrease in PetCO_2_ (Lucía et al., 2000). PPO was defined as the highest power output value reached during the test, and VO_2max_ was defined as the highest VO_2_ value (mean of 10 s) attained during the test.

#### 2.3.3 Muscle strength/power

Participants performed an incremental loading test on a Smith machine to assess muscle strength and power-related outcomes in the squat, lunge and hip-thrust exercises, as explained elsewhere (Gil-Cabrera et al., 2021; Valenzuela et al., 2021). Bar mean propulsive power (MPP) during the concentric phase was measured with a validated linear position transducer (T-Force System; Ergotech, Murcia, Spain) (Martínez-Cava et al., 2020). The initial weight was 20 kg (i.e., only the bar), and the load was increased by 10 kg until a decrease in MPP was observed in two consecutive loads. Participants performed three consecutive repetitions with each load, and a 2-minute rest was allowed between loads. We analyzed the highest MPP registered for each exercise and the one-repetition maximum (1RM) was estimated as explained elsewhere (Gil-Cabrera et al., 2021; Valenzuela et al., 2021).

#### 2.3.4 Time-trial

During the third visit, cyclists performed a simulated 8-minute time-trial after a standardized 10-minute warm-up at 60% of their PPO. Participants received no instructions regarding pacing and were blinded to power output values during the trial, but they were instructed to attain the highest mean power output possible, and they were allowed to adjust resistance by changing the gears of the bicycle. The mean power output during an 8-minute time trial has been reported as a valid indicator of performance in cyclists, being also correlated with laboratory-based performance indicators (e.g., lactate threshold) (Klika et al., 2007; Gavin et al., 2012; Sanders et al., 2020).

### 2.4 Statistical analysis

Data are presented as mean ± SD. Normality (Kolmogorov–Smirnov test) and homoscedasticity (Levene’s test) of the data were checked prior to any statistical treatment. Differences between age categories were performed by one-way analysis of variance. In order to reduce the risk of type I error, Bonferroni post-hoc tests were only conducted when a significant group effect was found. Effect sizes (Cohen’s d) were computed to examine the magnitude of the differences and considered small, moderate, large, very large and extremely large (d > 0.1, > 0.3, > 0.5, > 0.7 or > 0.9, respectively) (Hopkins et al., 2009) (Hopkins et al., 2009). Statistical analyses were performed with specific statistical software (SPSS 26.0, Inc., Chicago, IL, United States), setting the alpha for significance at 0.05.

## 3 Results

Differences between age categories for endurance, strength and body composition indicators are shown in Table 1, Table 2 and Table 3, respectively.

**TABLE 1:** Differences in endurance indicators between groups.

Variable	Professional	U23	Junior	Main *p*-value	ES Prof vs. U23	ES Prof vs. junior	ES U23 vs. junior
8-min TT (W)	396 ± 34	365 ± 29	322 ± 35	<0.001***	0.981*	2.144***	1.337**
8-min TT (W/kg)	6.06 ± 0.30	5.68 ± 0.44	5.30 ± 0.30	<0.001***	1.009*	2.533***	1.009*
PPO (W)	478 ± 37	446 ± 35	403 ± 41	<0.001***	0.888*	1.920***	1.128**
Relative PPO (W/kg)	7.31 ± 0.34	6.94 ± 0.44	6.64 ± 0.34	<0.001***	0.941*	1.970***	0.762
VO_2max_ (L/min)	5.49 ± 0.43	5.22 ± 0.47	4.62 ± 0.56	<0.001***	0.599	1.742***	1.160**
Relative VO_2max_ (ml/kg/min)	82.64 ± 3.54	79.79 ± 4.92	74.54 ± 3.55	<0.001***	0.664	2.284***	1.223**
Relative VO_2max_ (ml/kg MM/min)	95.28 ± 4.67	94.21 ± 5.09	88.24 ± 5.88	0.002**	0.219	1.325**	1.085**
PO at RCP (W)	414 ± 37	392 ± 40	339 ± 35	<0.001***	0.570	2.082***	1.410**
Relative PO at RCP (W/kg)	6.34 ± 0.30	6.10 ± 0.58	5.59 ± 0.49	0.001**	0.519	1.846***	0.949**
VO_2_ at RCP (%VO_2max_)	95.22 ± 2.87	95.90 ± 2.49	93.36 ± 2.07	0.018*	0.253	0.743	1.109*
PO at VT (W)	315 ± 34	266 ± 68	245 ± 27	<0.001***	0.911***	2.280***	0.405
Relative PO at VT (W/kg)	4.81 ± 0.37	4.15 ± 0.38	4.05 ± 0.42	<0.001***	1.759***	1.920***	0.249
VO_2_ at VT (%VO_2max_)	75.20 ± 4.57	70.55 ± 5.26	73.68 ± 6.05	0.031*	0.943*	0.283	0.552

Abbreviations: ES, effect size (Cohen’s d); PO, power output; PPO, peak power output; RCP, respiratory compensatory threshold; VO2max, peak oxygen uptake; MM, muscle mass; VT, ventilatory threshold. Significant differences: ^*^ *p* < 0.05;^**^ *p* < 0.01;^***^ *p* < 0.001.

**TABLE 2:** Differences in strength/power indicators between groups.

Variable	Professional	U23	Junior	Main *p*-value	ES Prof vs. U23	ES Prof vs. junior	ES U23 vs. junior
Squat 1RM (kg)	93.29 ± 20.83	87.17 ± 16.71	81.38 ± 12.89	0.206	0.324	0.687	0.387
Squat relative 1RM [kg/body mass (kg)]	1.41 ± 0.30	1.33 ± 0.24	1.33 ± 0.23	0.643	0.294	0.299	0.000
Squat MMP (W)	604 ± 175	556 ± 118	474 ± 76	0.036*	0.321	0.963*	0.826
Squat relative MMP [W/body mass (kg)]	9.07 ± 2.39	8.47 ± 1.59	7.71 ± 1.34	0.154	0.295	0.701	0.516
Hip thrust 1RM (kg)	113.64 ± 45.87	105.29 ± 29.54	107.85 ± 19.74	0.753	0.216	0.163	0.101
Hip thrust relative 1RM [kg/body mass (kg)]	1.70 ± 0.63	1.60 ± 0.40	1.74 ± 0.26	0.627	0.189	0.083	0.415
Hip thrust MMP (W)	472 ± 162	471 ± 126	504 ± 120	0.760	0.006	0.224	0.268
Hip thrust relative MMP [W/body mass (kg)]	7.07 ± 2.23	7.17 ± 1.73	8.15 ± 1.88	0.259	0.050	0.523	0.542
Split squat 1RM (kg)	51.50 ± 26.15	55.33 ± 18.85	54.31 ± 10.87	0.844	0.168	0.140	0.066
Split squat relative 1RM [kg/body mass (kg)]	0.77 ± 0.37	0.84 ± 0.26	0.88 ± 0.17	0.576	0.218	0.382	0.182
Split squat MMP (W)	285 ± 158	309 ± 106	273 ± 45	0.619	0.178	0.103	0.442
Split squat relative MMP [W/body mass (kg)]	4.25 ± 2.24	4.68 ± 1.44	4.43 ± 0.73	0.709	0.228	0.108	0.218

Abbreviations: 1RM, one-repetition maximum; ES, effect size (Cohen’s d); MMP, maximum mean power. Significant differences: ^*^ *p* < 0.05;^**^ *p* < 0.01;^***^ *p* < 0.001.

**TABLE 3:** Differences in body composition indicators between groups.

Variable	Professional	U23	Junior	Main *p*-value	ES Prof vs. U23	ES Prof vs. junior	ES U23 vs. junior
Height (cm)	177.03 ± 4.49	178.45 ± 5.40	176.08 ± 5.41	0.394	0.285	0.191	0.438
Body mass (kg)	66.56 ± 6.08	65.57 ± 5.93	61.87 ± 6.23	0.111	0.164	0.761	0.608
BMI (kg/m^2^)	21.20 ± 1.12	20.57 ± 1.27	19.94 ± 1.68	0.064	0.526	0.882	0.423
BMC (kg)	2.38 ± 0.28	2.41 ± 0.33	2.18 ± 0.25	0.080	0.098	0.753	0.785
BMC (%)	3.59 ± 0.31	3.67 ± 0.32	3.53 ± 0.24	0.356	0.253	0.216	0.494
Fat mass (kg)	6.50 ± 1.01	7.66 ± 1.26	7.42 ± 1.91	0.050	1.015	0.602	0.148
Fat mass (%)	9.78 ± 1.33	11.66 ± 1.36	11.90 ± 2.31	0.002**	1.025**	1.124**	0.126
Muscle mass (kg)	57.77 ± 5.50	55.49 ± 4.79	52.28 ± 4.87	0.023*	0.442	1.056*	0.664
Muscle mass (%)	86.77 ± 1.31	84.67 ± 1.30	84.58 ± 2.36	0.001**	1.609**	1.147**	0.047

Abbreviations: BMI, body mass index; BMC, bone mineral content; ES, effect size (Cohen’s d). Significant differences: ^*^ *p* < 0.05;^**^ *p* < 0.01;^***^ *p* < 0.001.

A significant group effect was observed for all endurance indicators, with a linear increase from juniors to professionals in most analyzed variables (Table 1). Accordingly, U23 and, particularly, professionals attained significantly higher values than juniors for most of the analyzed endurance indicators (i.e., 8-minute time-trial performance, VO_2max_, PPO, RCP, and VT). Differences between U23 and professionals were found only for the 8-minute time-trial, PPO and VT (all of them expressed in both absolute and relative units) and for the relative workload (%VO_2max_) at which the VT occurred. No differences were found for VO_2max_ or RCP. Of note, no consistent linear association (i.e., no increase from juniors to professionals) was observed for the relative workload (%VO_2max_) at which the VT and the RCP occurred.

Contrary to endurance indicators, no significant group effects were found for the analyzed strength indicators with the exception of the squat absolute MMP, which was higher in professionals than in juniors (Table 2). Likewise, no significant group effect was found for anthropometric variables such as body mass, BMI, BMC or absolute fat mass (Table 3). However, a significant group effect was found for relative fat mass—with professionals showing a significantly lower relative fat mass than both juniors and U23—as well as for absolute and relative muscle mass, both of which were higher among professionals than in U23 and, particularly, juniors.

## 4 Discussion

The main finding of the present study is that U23 and, particularly, professional cyclists differed from junior cyclists for most of the laboratory-based endurance indicators (e.g., VO_2max_, PPO, RCP, VT and simulated 8-minute time-trial performance). Similarly, there were evident anthropometric differences between professional cyclists and the other two groups, including lower relative fat mass and higher muscle mass levels. Finally, professional cyclists also had higher values for many endurance indicators compared with U23 cyclists, although no differences were found for major markers such as VO_2max_. Likewise, no consistent differences were found between age categories for strength/power indicators. Although longitudinal studies are warranted to confirm the influence of the abovementioned endurance and anthropometric indicators on performance, these findings might serve for performance prediction and talent identification, as well as to aid coaches in the design of training programs aimed at improving those variables that appear more determinant.

The utility of laboratory-based endurance indicators as determinants of cycling performance has been widely reported. In addition to the 8-minute time-trial performance, which can be considered an indicator of ‘actual’ performance—and indeed differed between all three age categories in the present study—most of the analyzed endurance indicators showed different results between junior cyclists and the remaining age categories, but only some indicators such as PPO (but not VO_2max_) differed between professionals and U23 cyclists. PPO, therefore, appears as a more sensitive marker of performance than VO_2max._ In line with this finding, Marín-Pagán et al. (2021) found that relative PPO was greater in U23 cyclists than in junior cyclists, with no differences in VO_2max_. Moreover, PPO has been reported to be more strongly correlated with cycling performance than VO_2max_ (Bishop et al., 1998, 2000; Bentley et al., 2001). In this line, Menaspà et al. (2012) reported that while VO_2max_ can discern between junior cyclists of different levels, its accuracy to predict future success (becoming professional) is limited. On the other hand, VT was also different between all three age categories, which is also in accordance with previous studies reporting stronger correlations between cycling performance and threshold-related parameters than for other indicators such as VO_2max_ (Nichols et al., 1997; Bishop et al., 1998, 2000; Bentley et al., 2001; Michalik et al., 2019). Thus, PPO and VT appear as more sensitive markers of performance (and a more sensitive differentiating factor between age categories) than VO_2max_ in well-trained or elite cyclists.

It is noteworthy that no linear association (i.e., no consistent increase from juniors to professionals) was observed for the relative workload (%VO_2max_) at which VT and RCP occurred. The fraction of VO_2max_ that can be sustained for a given period of time has been traditionally proposed as a marker of endurance performance (Joyner and Coyle, 2008), and it has been reported that trained subjects might have their lactate threshold (overall equivalent to ventilatory thresholds) at a higher %VO_2max_ than untrained peers (Joyner and Coyle, 2008), although this is controversial. In the present study U23 cyclists attained the RCP at a higher %VO_2max_ than juniors, but no differences were found between U23 and professionals. Similarly, professional cyclists attained the VT at a higher %VO_2max_ than U23 cyclists, but no differences were found between professionals and juniors, or between U23 and juniors. In line with these findings, a recent study reported that the fraction of %VO_2max_ at which the lactate threshold occurs was a poor individual predictor of performance (running velocity) and did not differentiate between elite, national and recreational runners (Støa et al., 2020). Therefore, the fraction of VO_2max_ at which thresholds occur should not be used individually as a predictor of endurance performance, unless it is used in combination with other parameters such as maximal aerobic speed or PPO (Støa et al., 2020).

Another major finding of the present study was that, although no differences in body mass were found between age categories, professional cyclists had a lower relative fat mass and higher levels of muscle mass than U23 and juniors. Previous studies have suggested that muscle mass indicators (e.g., quadriceps or thigh muscle cross-sectional area or volume) are positively associated with cycling performance, at least for short-duration efforts (Miura et al., 2002; Kordi et al., 2018, 2020a). In turn, a higher body mass can be negatively associated with performance (i.e., riding speed) owing to the influence of gravity, particularly on the steepest slopes. Thus, it seems that professional cyclists present with higher levels of muscle mass, which can be potentially associated with a greater power production capacity, but in turn a lower fat mass, which enables them to maintain an optimal body mass. Body composition appears, therefore, as a major differentiating factor between cyclists of different age categories.

Contrary to the findings for endurance indicators and body composition, no consistent differences between age categories were found for muscle strength/power indicators. A positive association between maximal strength and cycling performance has been previously reported, at least for short-duration efforts (Kordi et al., 2018, 2021). However, the association between strength capabilities and performance on longer efforts remains unclear. For instance, no associations have been found between critical power and knee extensor torque or back squat 1RM (Kordi et al., 2018; Byrd et al., 2021). Thus, although strength training has proven beneficial for cycling performance (Rønnestad et al., 2015, 2017; Kordi et al., 2020b), strength/power levels measured through an incremental loading test on a Smith machine in three different exercises seem not to be a major determinant of performance.

Some limitations of the present study should be noted. Despite the observed differences between groups, the cross-sectional design of the study precludes from drawing conclusions on performance prediction. Therefore, longitudinal studies are warranted to determine whether these variables could be used to accurately predict performance in young cyclists. Moreover, given that a large number of variables was assessed, the presence of type I error should not be disregarded. However, it must be noted that most variables would remain significant even when applying a stringent threshold *p*-value (i.e., *p* < 0.001). Finally, some methodological considerations should be also taken in mind. We used an incremental ramp protocol with short steps, which might influence the absolute and relative (%VO_2max_) position of ventilatory thresholds compared to longer steps (Bentley et al., 2007; Michalik et al., 2019). Moreover, the inclusion of a longer (e.g., 20 or 40 km) time trial, of other strength/power measures such as isometric strength tests (maximal torque and rate of force development) or Wingate anaerobic test, or other body composition indicators such as quadriceps’ cross-sectional area, could have provided additional information.

In summary, endurance indicators (e.g., VO_2max_, PPO, RCP, VT, simulated 8-minute time-trial performance) seem to be the main differentiating factor between junior cyclists and higher categories. Smaller, yet significant, differences in endurance indicators were also found between U23 and professional cyclists, with the latter showing higher time-trial performance, PPO and VT, but without differences in VO_2max_ and RCP. Some differences were also found in anthropometric parameters, with professional cyclists showing a lower relative fat mass and higher muscle mass compared with the remaining age categories. No consistent differences were found between age categories for strength/power indicators. Although longitudinal studies are needed to confirm these findings, the present results might help in performance prediction and/or talent identification and could also aid coaches in the design of training programs focused on improving those variables that appear more determinant.

## Data Availability

The raw data supporting the conclusion of this article will be made available by the authors, without undue reservation.
